# Joint hemorrhage accelerates cartilage degeneration in a rat immobilized knee model

**DOI:** 10.1186/s12891-020-03795-0

**Published:** 2020-11-19

**Authors:** Yasuhito Sogi, Yutaka Yabe, Yoshihiro Hagiwara, Masahiro Tsuchiya, Yoshito Onoda, Takuya Sekiguchi, Nobuyuki Itaya, Shinichiro Yoshida, Toshihisa Yano, Kazuaki Suzuki, Takahiro Onoki, Eiji Itoi

**Affiliations:** 1 Department of Orthopaedic Surgery, Tohoku University School of Medicine, 1-1 Seiryo-machi, Aoba-ku, Sendai, 980-8574 Japan; 2 Department of Nursing, Faculty of Health Science, Tohoku Fukushi University, 1-8-1 Kunimi, Aoba-ku, Sendai, 981-8522 Japan

**Keywords:** Cartilage degeneration, Joint hemorrhage, Joint immobilization, Mechanical stress

## Abstract

**Background:**

Joint hemorrhage is caused by trauma, ligament reconstruction surgery, and bleeding disorders such as hemophilia. Recurrence of hemorrhage in the joint space induces hemosiderotic synovitis and oxidative stress, resulting in both articular cartilage degeneration and arthropathy. Joint immobilization is a common treatment option for articular fractures accompanied by joint hemorrhage. Although joint hemorrhage has negative effects on the articular cartilage, there is no consensus on whether a reduction in joint hemorrhage would effectively prevent articular cartilage degeneration. The purpose of this study was to investigate the effect of joint hemorrhage combined with joint immobilization on articular cartilage degeneration in a rat immobilized knee model.

**Methods:**

The knee joints of adult male rats were immobilized at the flexion using an internal fixator from 3 days to 8 weeks. The rats were randomly divided into the following groups: immobilized blood injection (Im-B) and immobilized-normal saline injection (Im-NS) groups. The cartilage was evaluated in two areas (contact and non-contact areas). The cartilage was used to assess chondrocyte count, Modified Mankin score, and cartilage thickness. The total RNA was extracted from the cartilage in both areas, and the expression of metalloproteinase (*MMP*)-8, *MMP-13*, interleukin (*IL*)-1β, and tumor necrosis factor (*TNF*)-α was measured by quantitative real-time polymerase chain reaction.

**Results:**

The number of chondrocytes in the Im-B group significantly decreased in both areas, compared with that in the Im-NS group. Modified Mankin score from 4 to 8 weeks of the Im-B group was significantly higher than that of the Im-NS group only in the contact area. The expression of *MMP-8* and *MMP-13* from 2 to 4 weeks and *TNF-α* from 2 to 8 weeks significantly increased in the Im-B group compared with those in the Im-NS group, but there was no significant difference in *IL-1β* expression.

**Conclusions:**

The results showed that joint hemorrhage exacerbated immobilization-induced articular cartilage degeneration. Drainage of a joint hemorrhage or avoidance of loading may help prevent cartilage degeneration during joint immobilization with a hemorrhage.

**Supplementary Information:**

The online version contains supplementary material available at 10.1186/s12891-020-03795-0.

## Background

Joint hemorrhage is often caused by trauma, major joint surgery, and bleeding disorders, such as hemophilia. Repeated joint bleeding has negative effects on cartilage degeneration directly and indirectly [1, 2]. Iron accumulation in the joint cavity by hemorrhage induces free radical formation near the cartilage, causing chondrocyte apoptosis and matrix deformation [1, 3]. Moreover, iron induces excessive neovascularization and proliferation of synoviocytes [4], and the subsequent synovial inflammation leads to arthropathy [5]. Although this devastating event becomes more severe through repeated bleeding, it is induced by a single blood exposure [1, 5], which causes long-lasting impaired matrix turnover [5, 6] and joint damage progression.

In hemophilic arthropathy, pro-inflammatory cytokines such as interleukin (IL)-1β [6–8] and tumor necrosis factor (TNF)-α [6, 9] are produced in the hemosiderin-laden synovial tissue, and these cytokines trigger catabolic programs, activating nitric oxide (NO) [1, 6] and matrix metalloproteinase (MMP) expression [6], osteoarthritis [10], and rheumatoid arthritis. These cytokines also increase iron uptake into monocytes and synovial fibroblasts and accelerate the vicious cycle of synovitis [7]. However, methods that induce joint bleeding, including bone marrow stimulation, have been proven to be effective for treating cartilage defects of the knee through cartilage regeneration [11]. Additionally, a similar procedure was reported to enhance healing of the torn meniscus [12]. However, the beneficial effects have been shown only for damaged tissues, and its clinical outcome has been demonstrated only for a short term [7]. So far, the effects of hemorrhage on normal cartilage have not been considered.

Posttraumatic osteoarthritis after fracture or ligament injury occasionally occurs even with appropriate treatment or surgery [13]. According to previous studies, trauma and surgery can induce degeneration of the articular cartilage, inflammation, and hypertrophy of synovial cells, resulting in posttraumatic osteoarthritis [13, 14]. Additionally, joint immobilization, which is a common treatment for traumatic injury to maintain rest or enhance recovery, causes degenerative changes in the articular cartilage [15] and contributes to osteoarthritis development [16]. Blood in the joint is cleared rapidly within 48 h without joint immobilization [17]; however, a study reported protraction of residual blood in the joint-by-joint immobilization [18]. Thus, joint immobilization may exacerbate the harmful effect of blood on joint cartilage; although, this has not been investigated. Therefore, in this study, we investigated the effect of joint hemorrhage combined with joint immobilization on articular cartilage degeneration in a rat immobilized knee model.

## Methods

### Animals

Mature Sprague–Dawley male rats, aged 12 weeks old, were used (CLEA Japan Inc., Tokyo, Japan). The number of rats used in this study was determined in accordance with a previous study [19]. In total, 108 rats were used (histological and immunohistological evaluations, *n* = 72; gene expression analysis, *n* = 36). The protocol of this study was approved by the Institutional Animal Research Committee of Tohoku University before the experiments were initiated (approval number: 2013 MdA-360). The rats were subjected to anesthesia with intra-peritoneal administration of sodium pentobarbital (50 mg/kg) and their unilateral knee joint was rigidly immobilized at 150 degrees of the flexion with a plastic plate and two metal screws for various periods [19]. After surgery, the rats were randomly divided into the following groups: immobilized blood injection (Im-B) and immobilized-normal saline injection (Im-NS) groups. After surgery, a single injection of 50 μL autologous blood obtained from the caudal vein was administered directly into the knees of rats in the Im-B group [20]. An equal amount of normal saline was administered to rats in the Im-NS group in the same manner.

### Tissue preparation

The specimens were prepared for evaluation according to a previous study [21]. After euthanasia by intraperitoneal injection of sodium pentobarbital overdose, the rats were fixed with 4% paraformaldehyde (PFA) in 0.1 M phosphate buffered saline (PBS, pH 7.4) by perfusion into the aorta. Subsequently, the tissues around the knee joint were resected and maintained in the same fixative for 24 h at 4 °C. The fixed specimens were decalcified in 10% ethylenediaminetetraacetic acid (EDTA) for 2 months at 4 °C. After dehydration with ethanol and xylene solutions, the specimens were embedded in paraffin. They were cut into 5-μm sagittal sections in the medial midcondylar region of the knee and used for evaluation [21].

### Histological evaluation

Hematoxylin and eosin staining and Safranin O staining were performed to assess cartilage degeneration, chondrocyte count, and cartilage thickness in the contact and non-contact areas of the femur and tibia, respectively (1 and 3 days, and 1, 2, 4, and 8 weeks after surgery; *n* = 6/each group) [20]. Cartilage degeneration was evaluated using the Modified Mankin histological grading scheme [22]. The chondrocytes were counted within a predefined field of view, and cartilage thickness was defined as the distance between the cartilage surface and osteochondral junction in each area [20]. Synovitis (including pannus, synovial hyperplasia and sub-synovial inflammation) was evaluated using a synovial scoring system [23]. Additionally, hemosiderin deposition was evaluated by Perls’ Prussian blue staining (2, 4, and 8 weeks after surgery).

### Immunohistochemistry

The sections at each period were deparaffinized and soaked in 0.3% hydrogen peroxide. Cluster of differentiation 68 (CD68) was used as a marker of macrophage-like type A synoviocytes [24]. Endogenous peroxidase was inactivated with 3% H_2_O_2_ in PBS for 20 min at room temperature. The slides were incubated with mouse anti-rat CD68 antibody (MCA341 R, AbD Serotec, Raleigh, NC, USA; dilution, 1:400) for 24 h at 4 °C. Goat anti-mouse immunoglobulin (IgG) (Nichirei, Tokyo, Japan) for CD68 was used as the secondary antibody and incubated for 30 min at room temperature. The final detection step was carried out using 3,30-diaminobenzidine tetrahydrochloride (DAB) (Sigma, St. Louis, MO, USA) in 0.1 M imidazole and 0.03% H_2_O_2_ as the chromogen. Counterstaining was performed using Carazzi’s hematoxylin. For the negative control, normal mouse IgG (Dako, Copenhagen, Denmark) was used as the primary antibody. All slides were stained in one session. CD68-positive cells were enumerated at the posterior capsule including joint space and synovium [25].

### Gene expression analyses

After euthanasia, the cartilage in the contact and non-contact areas from the femur and tibia was obtained using a surgical knife and rongeur. The harvested cartilage samples were immediately immersed in 1 mL of QIAzol (Qiagen, Hilden, Germany). The samples were crushed and homogenized with a Polytron (Kinematica AG, Lucerne, Switzerland). The total RNA was extracted using the RNeasy Lipid Tissue Mini Kit (Qiagen). Complementary DNA (cDNA) was synthesized using the Cloned AMV First-strand cDNA Synthesis Kit (Invitrogen, Carlsbad, CA, USA). The efficiency of polymerase chain reaction (PCR) and relative expression levels of *MMP-8*, *MMP-13*, *IL-1β*, and *TNF-α* as a function of elongation factor-1α1 (EF1α1) were calculated as described previously (2, 4, and 8 weeks after surgery; *n* = 6/each group) [26]. The primer sequences used in the PCR are listed in Table 1.

**Table 1:** Sequence of Primers Used for Polymerase Chain Reaction

Gene Name	GenBank	Nucleic Acid Sequence	
*MMP-8*	NM 022221	Upstream Downstream	tggaatccttgcccatgcct aagcagccacgagaaacaggt
*MMP-13*	NM 133530	Upstream Downstream	tggtcttctggcacaacgctt tggaagctgcttgtccaggtt
*IL-1β*	NM 031512	Upstream Downstream	accgtggcaacattctggtca tcgacaaatgctgcctcgtga
*TNF-α*	NM 0126763	Upstream Downstream	actggcgtgttcaatccgttctctac ccgcaaaatccaggccactacttc
*EF1a1*	NM 175838	Upstream Downstream	tgatgccccaggacacaagagaact gataccagcttcaaaattcccaacaac

### Statistical analyses

Differences between the Im-B and Im-NS groups were assessed using unpaired *t*-test (histological evaluation: Mankin score, chondrocyte count, and cartilage thickness, CD68-positive cell count) and Mann–Whitney’s *U* test (RT-qPCR). Data are presented as mean ± standard deviation (SD). Tests were two-sided, and a value of *p* < 0.05 indicated statistical significance. The inter-observer variabilities for Mankin score, chondrocyte count, and cartilage thickness were assessed using intraclass correlation coefficients, which were 0.92, 0.62, and 0.82, respectively. All statistical analyses were performed using IBM SPSS Statistics version 24.0 (SPSS Japan Inc., Tokyo, Japan).

## Results

### Histological evaluation

**Fig. 1:**
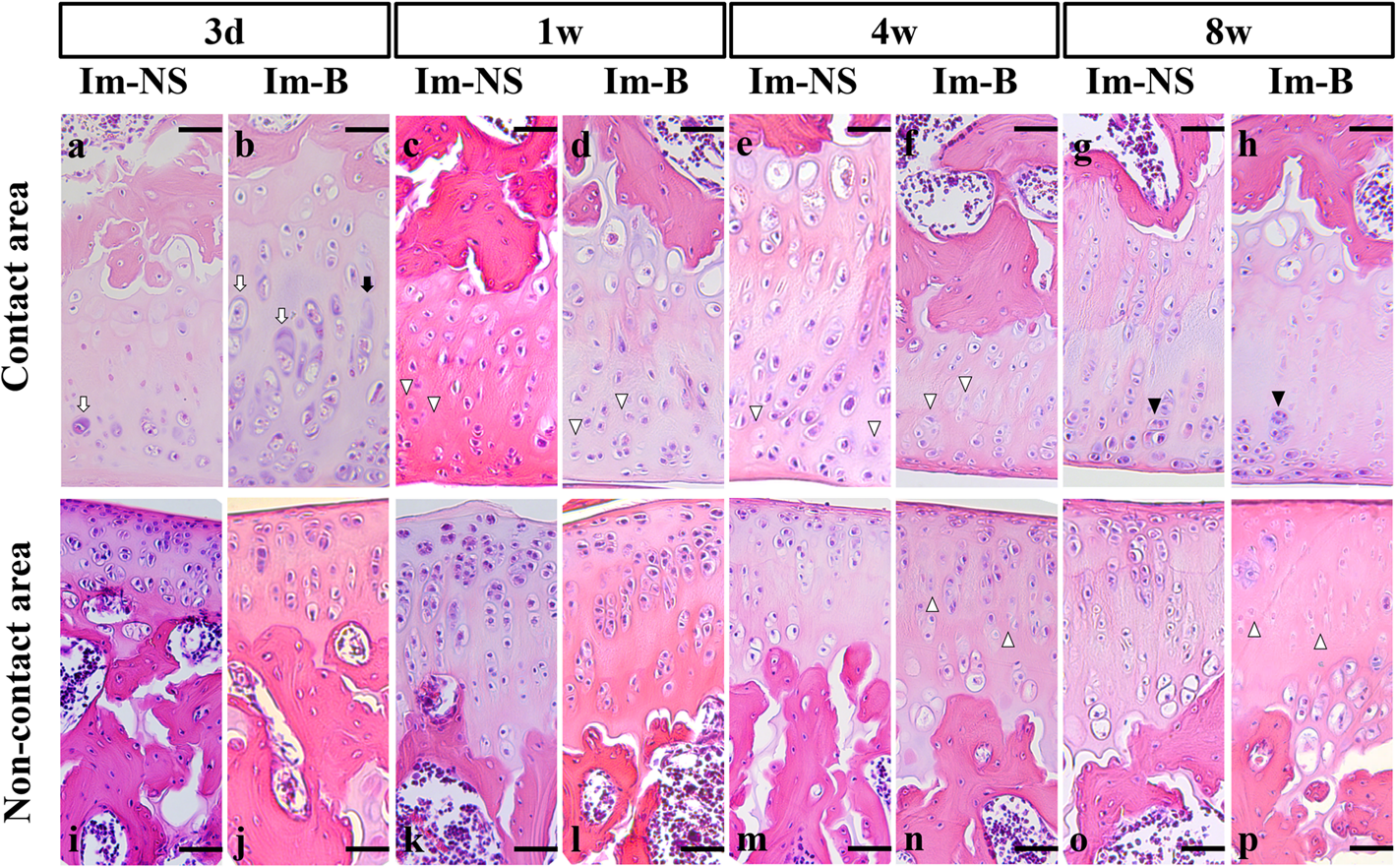
Histological features of femoral articular cartilage. The changes were observed after immobilization and intra-articular administration of blood (Im-B) or normal saline (Im-NS) from 3 days to 8 weeks. Upper row (**a-h**) showing the changes in cartilage in the contact area. Cartilage degeneration and decreasing number of chondrocytes gradually progressed after immobilization. Degeneration of the non-contact area is shown in the lower row (**i-p**). Although similar cellularity was observed in the contact and non-contact areas, cartilage degeneration was milder in the non-contact areas. Hematoxylin and eosin staining. The white arrows indicate the swelling of chondrocytes and the black arrows indicate the disappearance of the cells. The white arrowheads indicate cells with decreased cell staining intensity. The black arrowheads indicate cloning cells. Scale bar = 50 μm

Histological features of the hematoxylin and eosin-stained femoral cartilage are shown in Fig. 1. Articular cartilage degeneration was observed in both Im-B and Im-NS groups. The degeneration progressed gradually after immobilization, and it was more significant in the contact area (Fig. 1 a-h). At 3 days after immobilization and intra-articular administration, swelling of the chondrocytes and disappearance of cells in the tangential zone were observed, especially in the Im-B group (Fig. 1a and b). The cell layers were irregular at 1 week (Fig. 1c and d). The intensity of cell staining, which was more severe in the Im-B group, decreased at 4 weeks (Fig. 1e and f). Cartilage surface irregularity was observed at 2 weeks in the Im-B group (Supplementary Fig. 1). Cell cloning and cell loss occurred at 8 weeks (Fig. 1g and h). Degeneration of the non-contact area is shown in the lower row of Fig. 1(i-p). Although a similar cellularity was observed in the contact and non-contact areas, cartilage degeneration was milder in the non-contact areas than in the contact areas. A reduction in Safranin O staining intensity was observed at 8 weeks in both groups, and it was more severe in the Im-B group than in the Im-NS group (Supplementary Fig. 2).

**Fig. 2:**
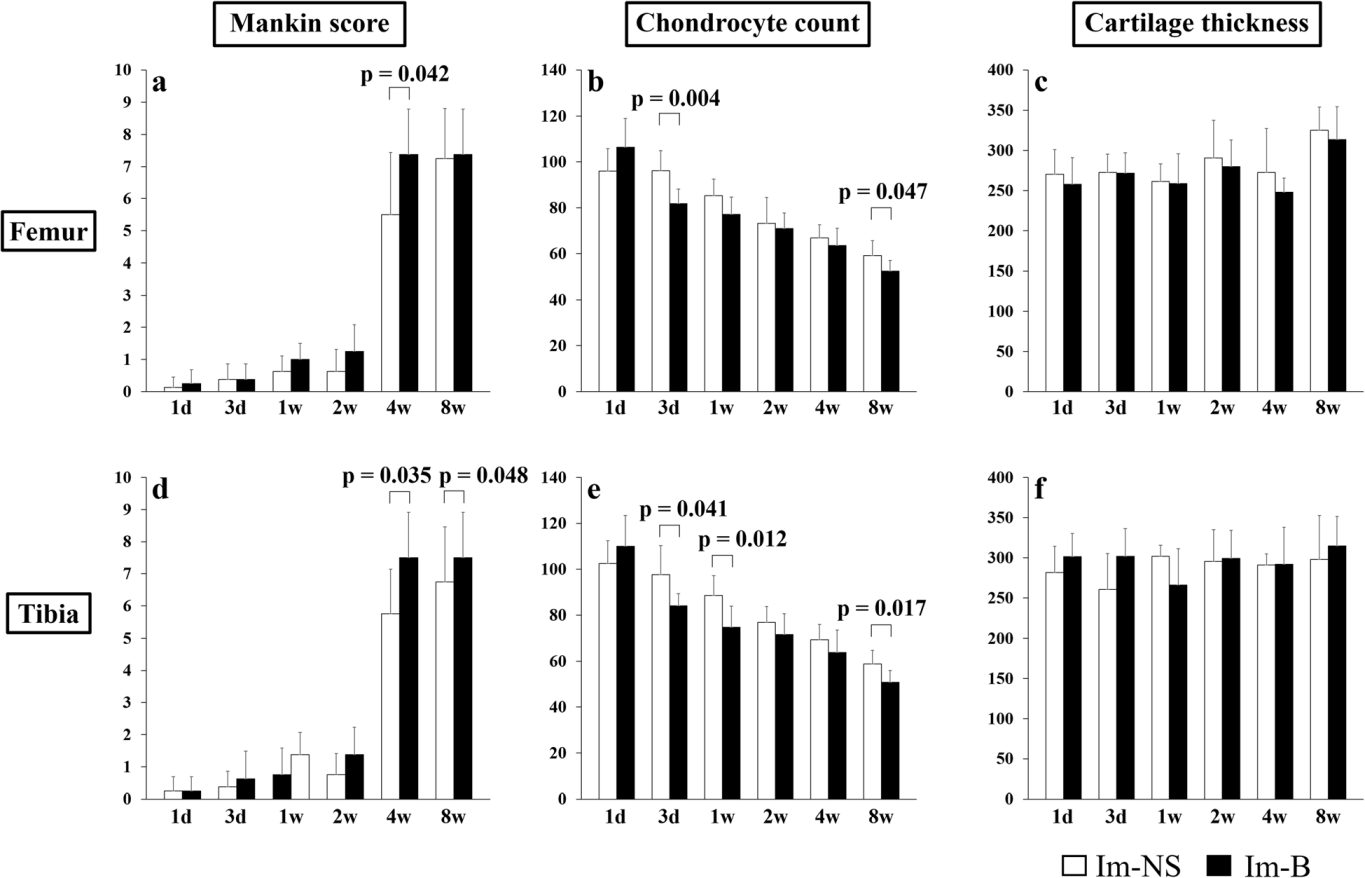
Time-lapse changes in the femoral and tibial cartilage in the contact area. Mankin score of the Im-B group was significantly higher than that of the Im-NS group at 4 weeks in the femurs and 4 and 8 weeks in the tibias (**a** and **d**). The number of chondrocytes in the Im-B group significantly decreased at 3 days and 8 weeks in the femur and 3 days and 1 and 8 weeks in the tibia compared with that in the Im-NS group (**b** and **e**). Cartilage thickness did not change and was not affected by immobilization and intra-articular administration (**c** and **f**, Fig. 3c and f)

**Fig. 3:**
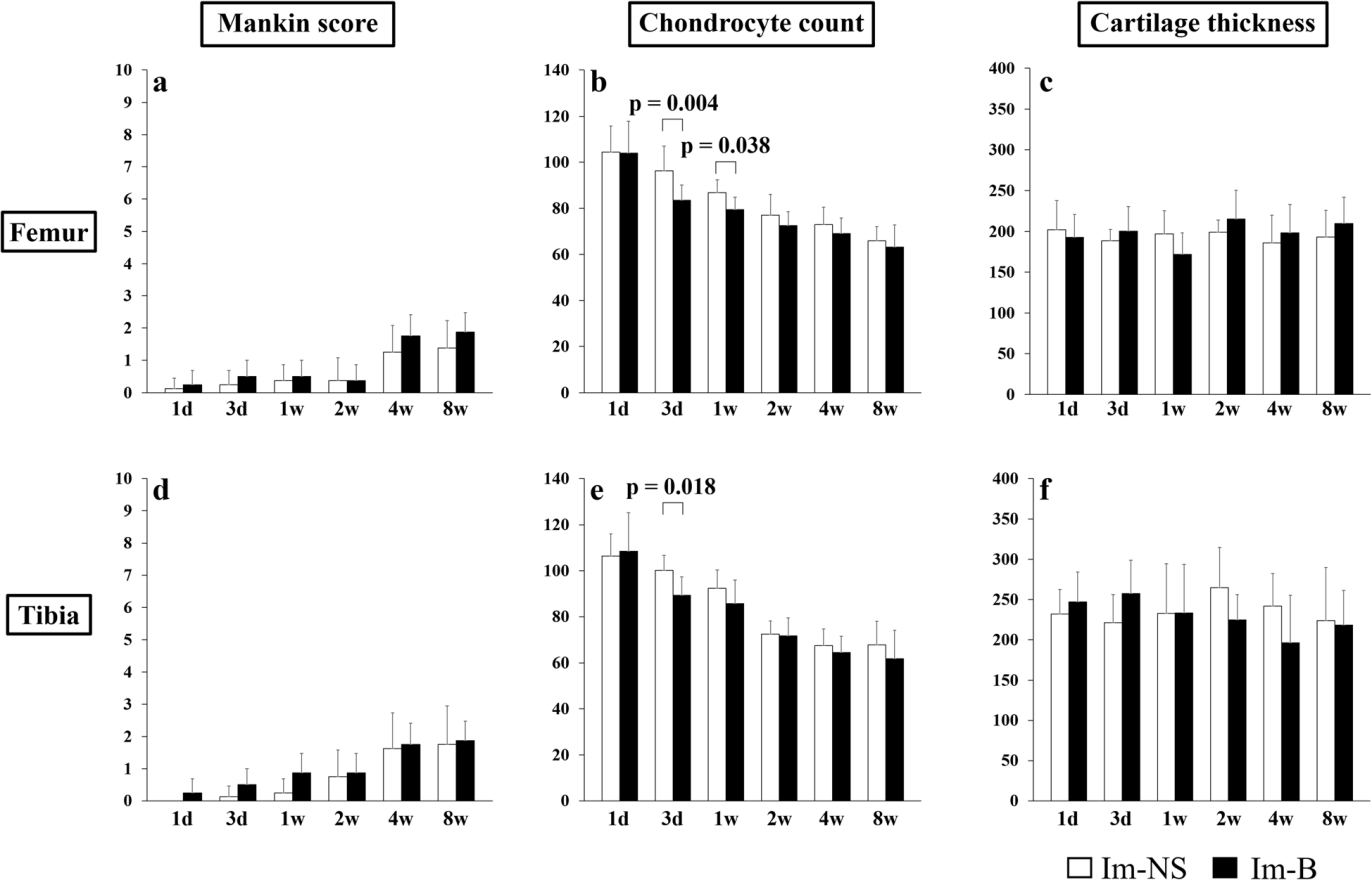
Time-lapse changes in the femoral and tibial cartilage in the non-contact area. Mankin score did not change significantly, and there was no statistical difference between the Im-B and Im-NS groups (**a** and **d**). The number of chondrocytes in the Im-B group significantly decreased at 3 days and 1 week in the femur and 3 days in the tibia (**b** and **e**). Cartilage thickness did not change, and it was not affected by immobilization and intra-articular administration in both contact and non-contact areas (**c** and **f**)

Mankin score of the Im-B group was significantly higher than that of the Im-NS group at 4 weeks in the femur, and 4 and 8 weeks in the tibia (Fig. 2a and d). There was no significant change in the non-contact area, and the difference between the Im-B and Im-NS groups was not significant (Fig. 3a and d). The number of chondrocytes in the contact area of the Im-B group significantly decreased at 3 days and 8 weeks in the femur and 3 days and 1 and 8 weeks in the tibia compared with that in the Im-NS group (Fig. 2b and e). In the non-contact area of the Im-B group, the number of chondrocytes significantly decreased at 3 days and 1 week in the femur and 3 days in the tibia (Fig. 3b and e). Cartilage thickness did not change, and it was not affected by immobilization and intra-articular administration in both contact and non-contact areas (Fig. 2c and f, Fig. 3c and f). The synovitis score of the Im-B group was slightly but not significantly higher than that of the Im-NS group at all periods (Supplementary Fig. 3).

**Fig. 4:**
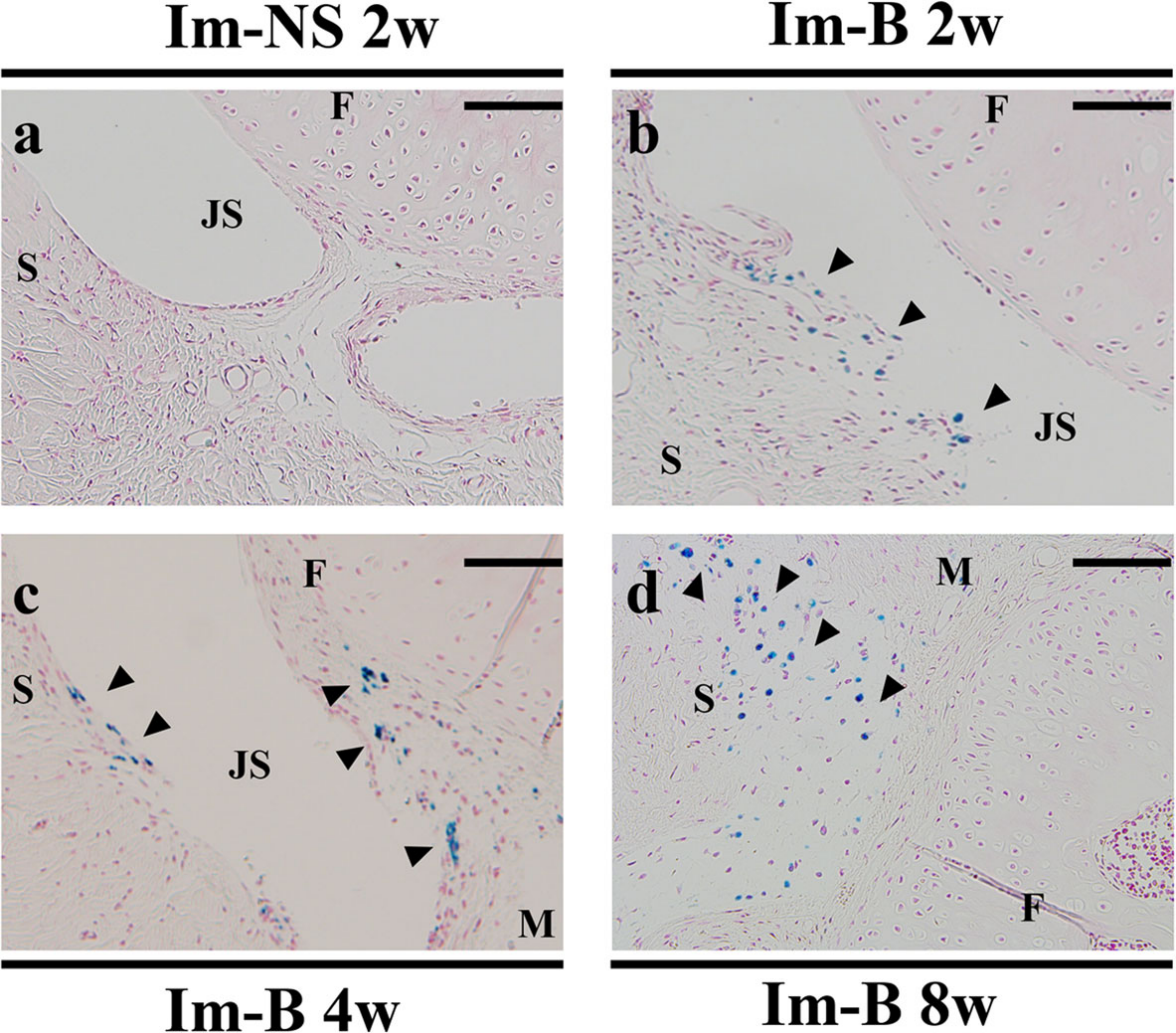
Iron deposition in the synovial membrane and meniscal surface. There was no iron deposition in the Im-NS group (**a**). Iron deposition was observed in the synovium and on the meniscal surface, lasting until 8 weeks after administration in the Im-B group (**b**-**d**). F: Femur, S: Synovium, JS: Joint Space, M: Meniscus. Perls: Prussian blue staining. The black arrowheads indicate iron depositions. Scale bar = 100 μm

**Fig. 5:**
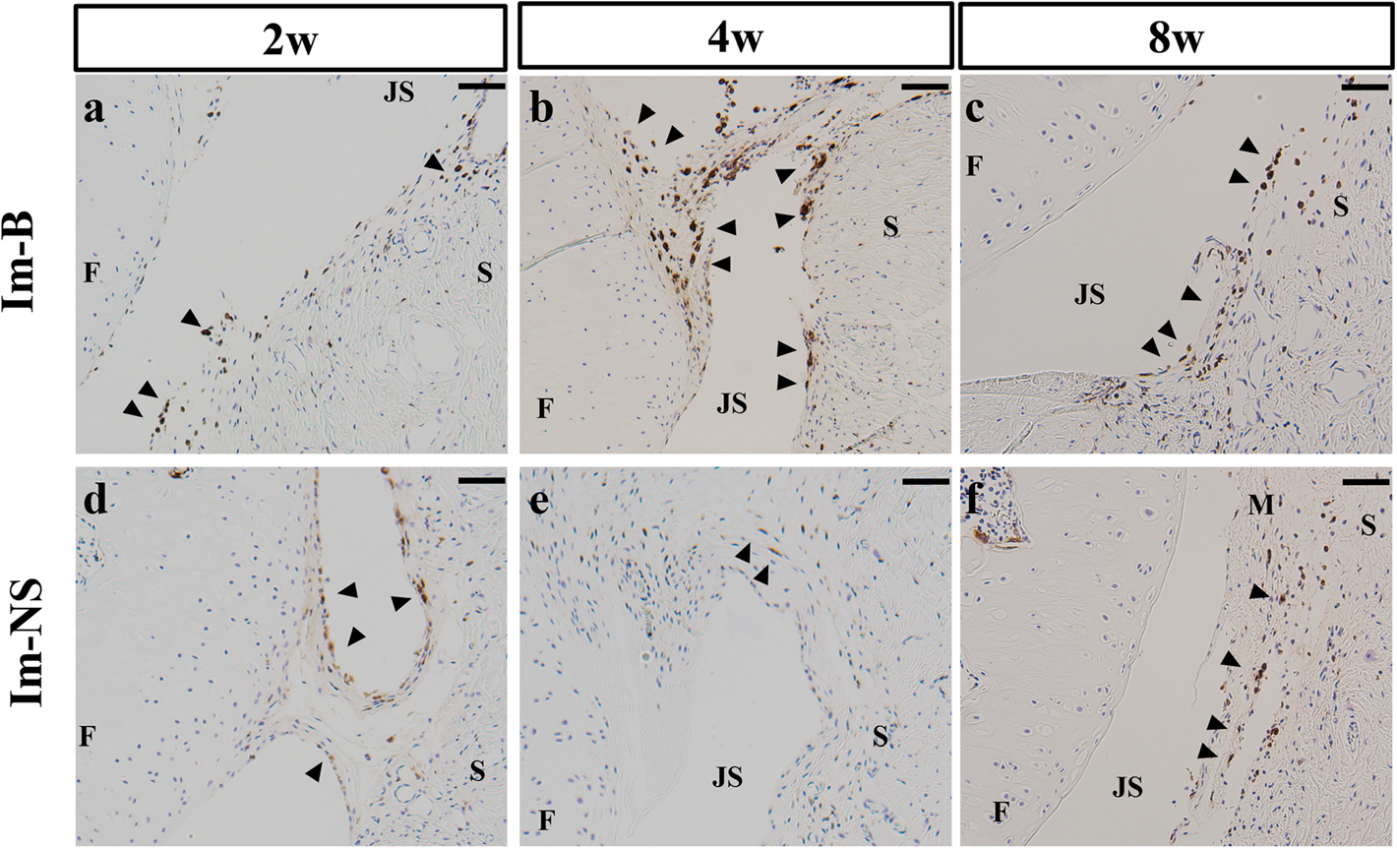
CD68 immunostaining in the Im-B and Im-NS groups. CD68-positive cells were observed in both Im-B (**a**-**c**) and Im-NS groups (**d**-**f**). They were observed mainly in the synovial membrane of the Im-B group. After 8 weeks, there was a small difference in the number of CD68-positive cells between the two groups. F: Femur, S: Synovium, JS: Joint Space, M: Meniscus. The black arrowheads indicate CD68-positive cells. Scale bar = 100 μm

Iron deposition evaluated by Perls’ Prussian blue staining is shown in Fig. 4. The injection of blood could induce joint bleeding, but there was no iron deposition in the Im-NS group (Fig. 4a). Iron deposition was observed in the synovium and meniscal surface, which lasted until 8 weeks after the administration of blood in the Im-B group (Fig. 4b-d). CD68-positive cells were observed in both Im-B and Im-NS groups, and primarily, in the synovial membrane (Fig. 5). The number of CD68-positive cells was slightly but not significantly higher at 4 weeks in Im-B group and 8 weeks in Im-NS group (Supplementary Fig. 4).

### Gene expression analyses

**Fig. 6:**
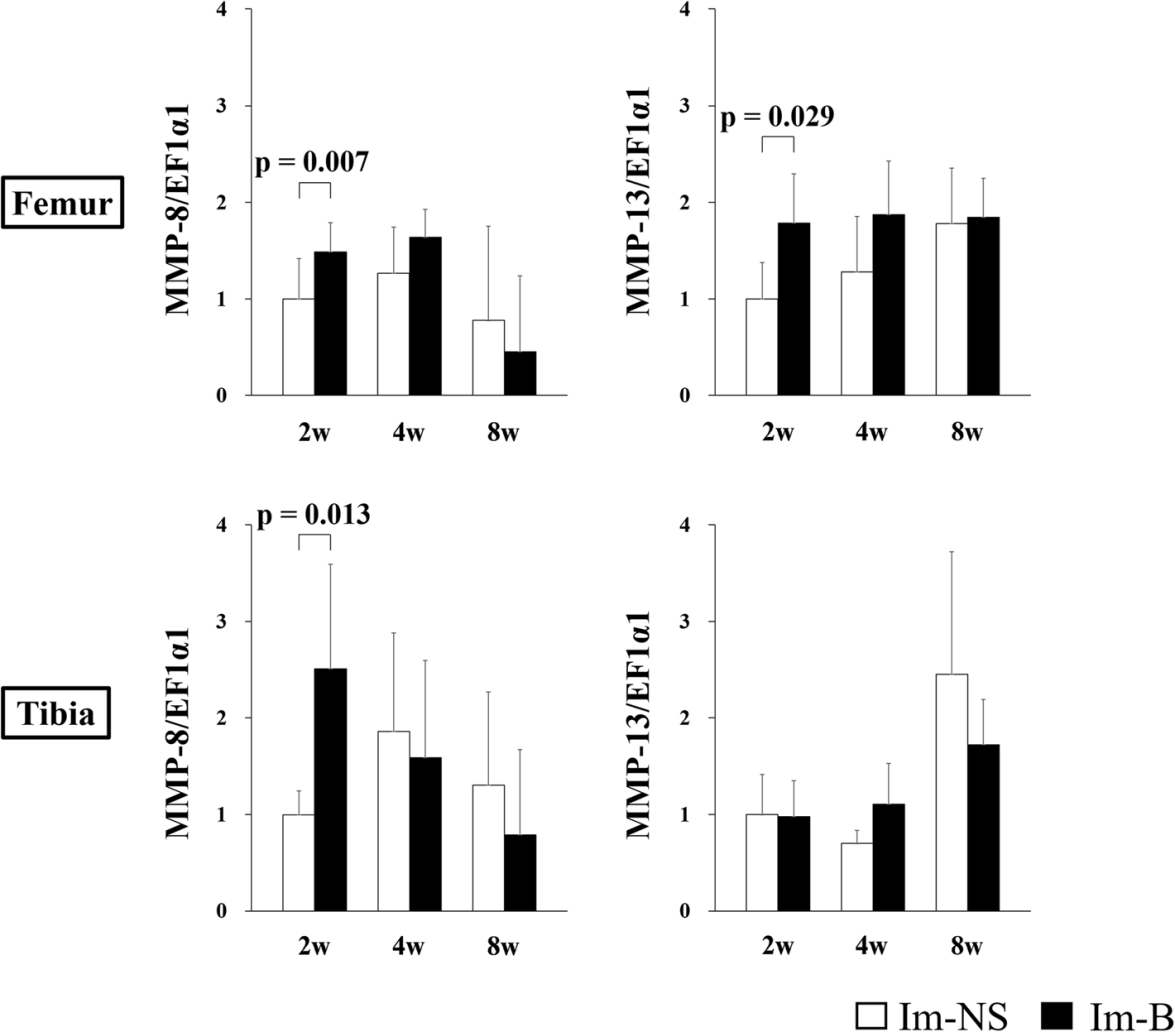
Expression of matrix metalloproteinase (*MMP*)-8 and *MMP-13* in the cartilage in the contact area. *MMP-8* and *MMP-13* expression at 2 weeks in the femur and *MMP-8* expression at 2 weeks in the tibia were significantly increased in the Im-B group compared with those in the Im-NS group

**Fig. 7:**
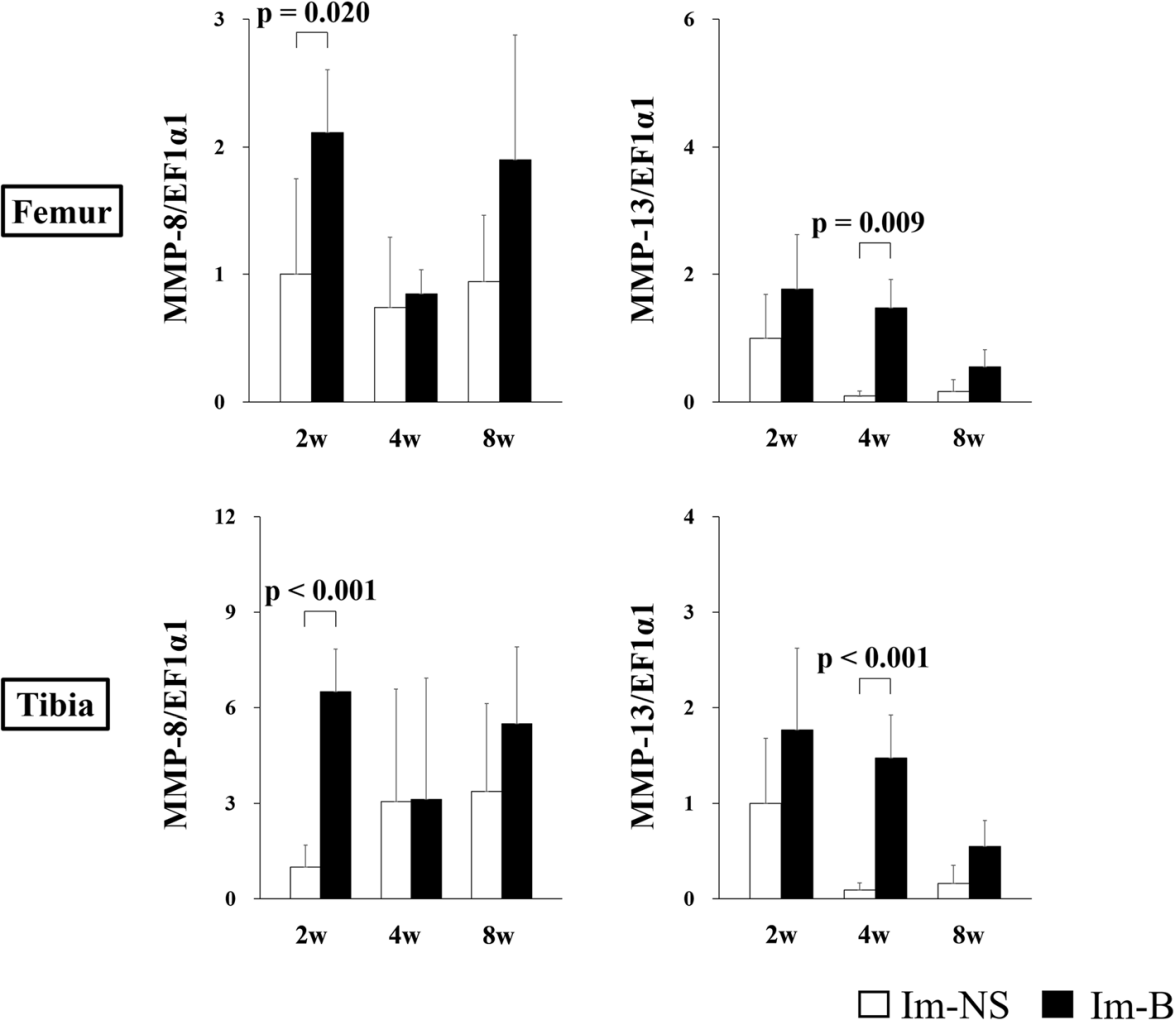
Expression of matrix metalloproteinase (*MMP*)-8 and *MMP-13* in the cartilage in the non-contact area. *MMP-8* expression at 2 weeks and *MMP-13* expression at 4 weeks in both femur and tibia were significantly increased in the Im-B group compared with those in the Im-NS group

**Fig. 8:**
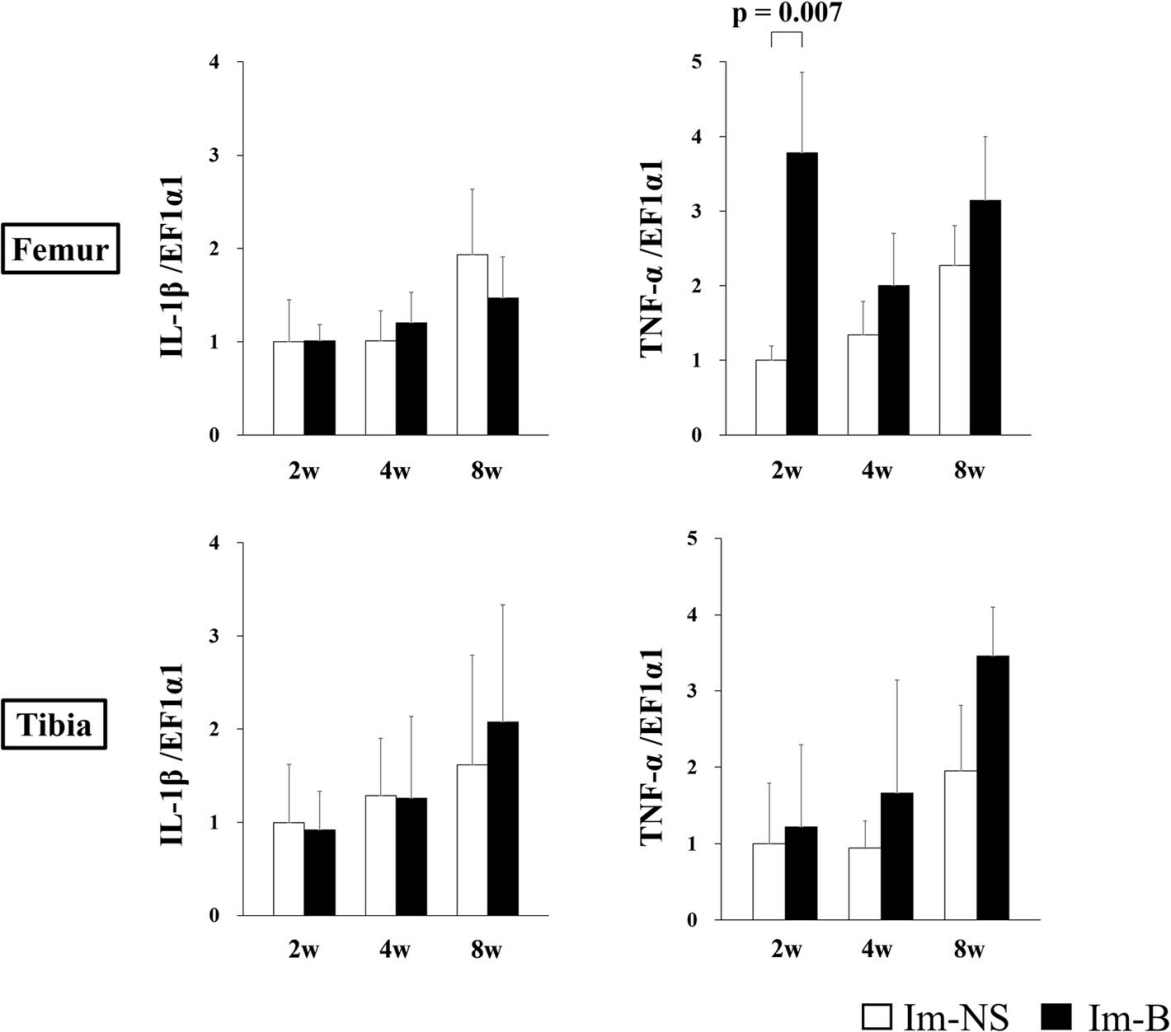
Interleukin-1β (*IL-1β*) and tumor necrosis factor-α (*TNF-α*) expression in the cartilage in the contact area. There was no significant difference in *IL-1β* expression. *TNF-α* expression in the femur was significantly increased in the Im-B group compared with that in the Im-NS group at 2 weeks

**Fig. 9:**
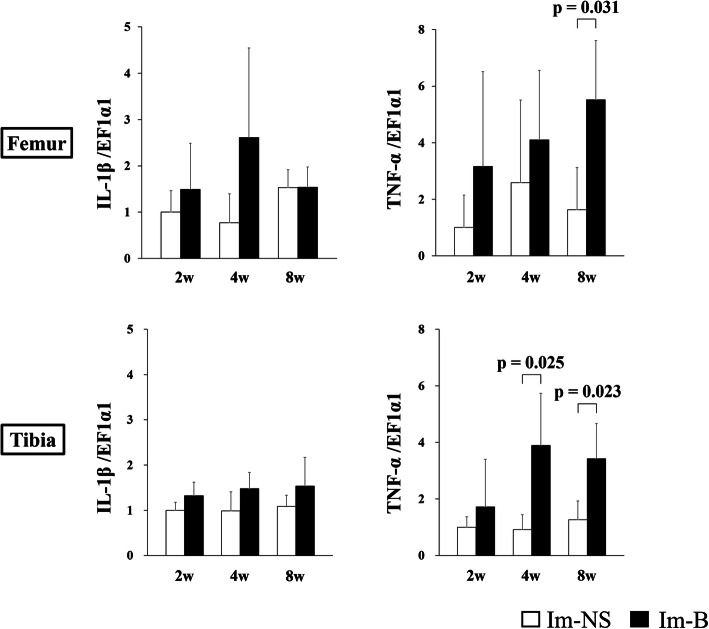
Interleukin-1β (*IL-1β*) and tumor necrosis factor-α (*TNF-α*) expression in the cartilage in the non-contact area. There was no significant difference in *IL-1β* expression. *TNF-α* expression in the femur at 8 weeks and tibia at 4- and 8- weeks was significantly increased in the Im-B group compared with that in the Im-NS group

The expression of genes related to collagenase (*MMP-8* and *MMP-13*) in the cartilage of the contact area is shown in Fig. 6. The expression of *MMP-8* and *MMP-13* in the femur at 2 weeks and that of *MMP-8* in the tibia at 2 weeks were significantly increased in the Im-B group compared with those in the Im-NS group. *MMP-8* and *MMP-13* expression in the non-contact area is shown in Fig. 7. The expression of *MMP-8* at 2 weeks and *MMP-13* at 4 weeks in both femur and tibia was significantly increased in the Im-B group compared with that in the Im-NS group. The expression of inflammation-related genes (*IL-1β* and *TNF-α*) in the cartilage in the contact and non-contact areas is shown in Figs. 8 and 9. There was no significant difference in *IL-1β* expression. *TNF-α* expression in the femoral contact area was significantly increased in the Im-B group compared with that in the Im-NS group at 2 weeks. In contrast, its expression in the non-contact area of the femur at 8 weeks and tibia at 4 and 8 weeks was significantly increased in the Im-B group compared with that in the Im-NS group.

## Discussion

Our study demonstrated that joint hemorrhage exacerbated articular cartilage degeneration in a rat immobilized knee model. Joint hemorrhage progression decreased the number of chondrocytes, independent of the cartilage area. Iron deposition in and inflammation of the synovium persisted until 8 weeks after blood injection, and joint hemorrhage increased the expression of *MMP-8* and *MMP-13* throughout the cartilage. Furthermore, joint hemorrhage aggravated cartilage degeneration induced by joint immobilization in the contact area. The differences in the contact and non-contact areas of the immobilized knee model can be attributed to the degree of mechanical stress or residual mobility [15, 27, 28]. Patellar mobility was not restricted, and it could move slightly in the medial or lateral direction. In the femoral non-contact area adjoined to the patella, immobility rigidity was less than that in the contact area.

Mechanical stress on cartilage determines the delicate balance between cartilage growth and breakdown [29]. Some studies have reported that mechanical stress induces cartilage degeneration, whereas others have shown that mild and moderate loading can stimulate cartilage matrix synthesis [29–31]. Joint hemorrhage, when combined with mechanical loading, exerts harmful effects on cartilage matrix turnover and integrity [32]. In our immobilized knee model, significant decreases in the number of chondrocytes were observed in both contact and non-contact areas of the Im-B group. However, cartilage degeneration was severe only in the contact area of the Im-B group. A previous study showed that iron-induced synovitis and hydroxy radicals cause chondrocyte apoptosis [7], but we did not assess it. The results of this study suggested that chondrocyte death by apoptosis or necrosis was stimulated by hemorrhage, regardless of mechanical stress. Thus, avoidance of mechanical stress may prevent the progression of cartilage degeneration. In clinical practice of hemophilic arthropathy treatment, articular cartilage is damaged symmetrically and broadly [8]. However, there have been no studies to evaluate the changes in cartilage thickness due to joint hemorrhage. A previous study assessed cartilage thickness using a rat immobilized knee model [15] and reported that joint immobilization induced cartilage thickening due to cartilage regeneration induced by compressive and shear force at the joint. In this study, cartilage thickness was not altered by joint hemorrhage and immobilization. The difference might be due to the dual effects of cartilage regeneration and degeneration caused by joint immobilization and hemorrhage, immobilization periods, or portion of cartilage assessed in the study.

Iron deposition was observed for up to 8 weeks in the Im-B group. Jansen et al. described opsonization of injected red blood cells into the joint cavity and recognition as foreign by macrophages and synoviocytes [17]. Additionally, the concentration of red blood cells in the joint cavity decreased to less than 5% within 48 h; however, this time course was sufficient to adversely affect the cartilage and synovial tissues [17]. Furthermore, Onoda et al. reported iron deposition for 8 weeks in the synovial membrane and capsule, employing the same model used in this study [18]. The results were consistent with our findings, indicating that immobilization could inhibit hemosiderin absorption and prolong hemosideric inflammation. The synovitis score was slightly but not significantly higher in the Im-B group than in the Im-NS group, and CD68-positive cells were primarily observed in the synovial membrane, indicating that iron deposition potentially induces synovial inflammation. Furthermore, CD68-positive cells tended to be more numerous in the Im-B group than in the Im-NS group at 4 weeks, and this number was similar in both groups at 8 weeks. The effect of joint hemorrhage on inflammation reduced after 4 weeks, after which immobilization was considered as the main cause of inflammation [25, 33].

MMP-8 and MMP-13 act as collagenases to cleave type II collagen, which is the basis of articular cartilage [34]. MMP-8 is expressed in neutrophils, osteoarthritic chondrocytes, articular chondrocytes, and synovial fibroblasts [35]. It interacts with inflammatory cytokines and contributes to chronic inflammatory diseases. In contrast, MMP-13 is the only collagenase implicated in the degradation of collagenous matrices, and has a higher enzyme activity than other MMPs in osteoarthritis [36]. MMP-13 is known to be involved in hemophilic arthropathy and rheumatoid arthritis [3]. Our study revealed that joint hemorrhage significantly increased the expression of *MMP-8* and *MMP-13* compared with that in the control group, regardless of mechanical stress. The findings indicated that even a single joint hemorrhage could lead to a higher expression of *MMP-8* and *MMP-13*, which can cause cartilage breakdown. *MMP-13* expression increased at 2 weeks in the contact area and at 4 weeks in the non-contact area of the Im-B group compared with that in the Im-NS group. Mechanical stress and strain have been associated with *MMP-13* activation and synthesis [37, 38], and the difference in the time of expression in our study could be due to the influence of mechanical stress.

There are numerous studies on inflammatory cytokines such as IL-1α, IL-1β, and TNF-α in joint disorders, including osteoarthritis [10], rheumatoid arthritis, posttraumatic osteoarthritis [14], and hemophilic arthropathy [2, 6]. These cytokines are detected in the synovial fluid, synovial membrane, and cartilage [39], affecting chondrocytes and resulting in tissue destruction [10]. It is considered that TNF-α promotes acute inflammation, whereas IL-1 plays a pivotal role in sustaining inflammation and cartilage destruction [40]. IL-1β and TNF-α regulate MMP activation [38], and the activation of IL-1β and TNF-α originally precedes that of MMPs [41]. In our study, *TNF-α* expression in the contact area was higher in the Im-B group than the Im-NS group at 2 weeks, which could lead to the subsequent MMP expression.

Mechanical stress activates TNF-α expression [42]. Joint hemorrhage also increases *TNF-α* expression [6, 41], which might strengthen the effect of mechanical stress [41]. Conversely, *TNF-α* expressions in the non-contact area was higher in the Im-B group than in the Im-NS group at 4 or 8 weeks. It might be due to chronic inflammation induced by prolonged deposition of hemosiderin without mechanical stress [42]. Additionally, there was no significant difference in *IL-1β* expression between the hemorrhage and control groups. We speculate that the effect of immobilization on IL-1β gene expression might exceed that of hemorrhage.

The results of this study may have clinical implications. Joint hemorrhage exacerbated cartilage degeneration induced by joint immobilization. Drainage of a joint hemorrhage or avoidance of loading may help prevent cartilage degeneration during joint immobilization with a hemorrhage, and this should be assessed in future studies.

This study had some limitations. First, the actual amount of blood administered into the joint was not assessed. Second, we did not quantify protein expression related to the pathogenesis. Third, gene expression was not evaluated within 2 weeks. Fourth, the synovium was not assessed by PCR, and therefore, we could not show the active synovitis and its influence on cartilage. Fifth, the number of rats was not determined on the basis of the statistical power. Finally, we did not include a normally loaded control with blood injection, which made the difference between the effects of hemorrhage with or without loading unclear; this necessitates further studies.

## Conclusions

Joint hemorrhage exacerbated cartilage degeneration induced by joint immobilization. Drainage of a joint hemorrhage or avoidance of loading may help prevent cartilage degeneration during joint immobilization with a hemorrhage.

## Supplementary Information

**Additional file 1:** **Figure S1.** Histological features of the femoral and tibial cartilage surface at 2 weeks in Im-B and Im-NS groups. The cartilage surface was smooth in the Im-NS group (a), irregularity of the femoral and tibial cartilage surface was observed in Im-B group (b). F: Femur, S: Synovium, JS: Joint Space, M: Meniscus. The black arrowheads indicate cartilage irregularity. Scale bar = 500 μm.

**Additional file 2:** **Figure S2.** Safranin O staining of the femoral articular cartilage. The changes were observed after immobilization and intra-articular administration of blood (Im-B) or normal saline (Im-NS) at 1- and 8- weeks. The upper row (a-d) shows the changes in the cartilage in the contact area and the lower row (e-h) shows the changes in the cartilage in the non-contact area. A reduction in Safranin O staining intensity was not observed at 1 week in the contact and non-contact areas (a, b, e, and f). A reduction in staining intensity was observed at 8 weeks in the contact area, and the reduction in the Im-B group was more severe than that observed in the Im-NS group (c and d). A similar reduction in staining was observed in the non-contact area, and there were no significant differences between the Im-B and Im-NS groups (g and h). The white arrowheads indicate a reduction in staining intensity at the extracellular matrix. Scale bar = 50 μm.

**Additional file 3:** **Figure S3.** The histological scoring of synovitis from 2 to 8 weeks. The synovitis score of the Im-B group was slightly but not significantly higher than that of the Im-NS group at all periods.

**Additional file 4:** **Figure S4.** The number of CD68-positive cell from 2 to 8 weeks. The number of CD68-positive cells increased slightly but not significantly at 4 weeks in Im-B group and 8 weeks in Im-NS group.

## Data Availability

The datasets used and/or analyzed during the current study are available from the corresponding author on reasonable request.

## Abbreviations

Im-B: Immobilized blood injection
Im-NS: Immobilized-normal saline injection
MMP: Metalloproteinase
IL: Interleukin
EF1α1: Elongation factor-1α1
TNF: Tumor necrosis factor
NO: Nitric oxide
PFA: Paraformaldehyde
EDTA: Ethylenediaminetetraacetic acid
CD: Cluster of differentiation
IgG: Immunoglobulin G
DAB: Diaminobenzidine tetrahydrochloride
cDNA: Complementary DNA
