# Supplementary figures and images for: Joint hemorrhage accelerates cartilage degeneration in a rat immobilized knee model
Source: BMC Musculoskelet Disord. 2020 Nov 19;21:761. doi: 10.1186/s12891-020-03795-0 (PMC7678279; doi:10.1186/s12891-020-03795-0)

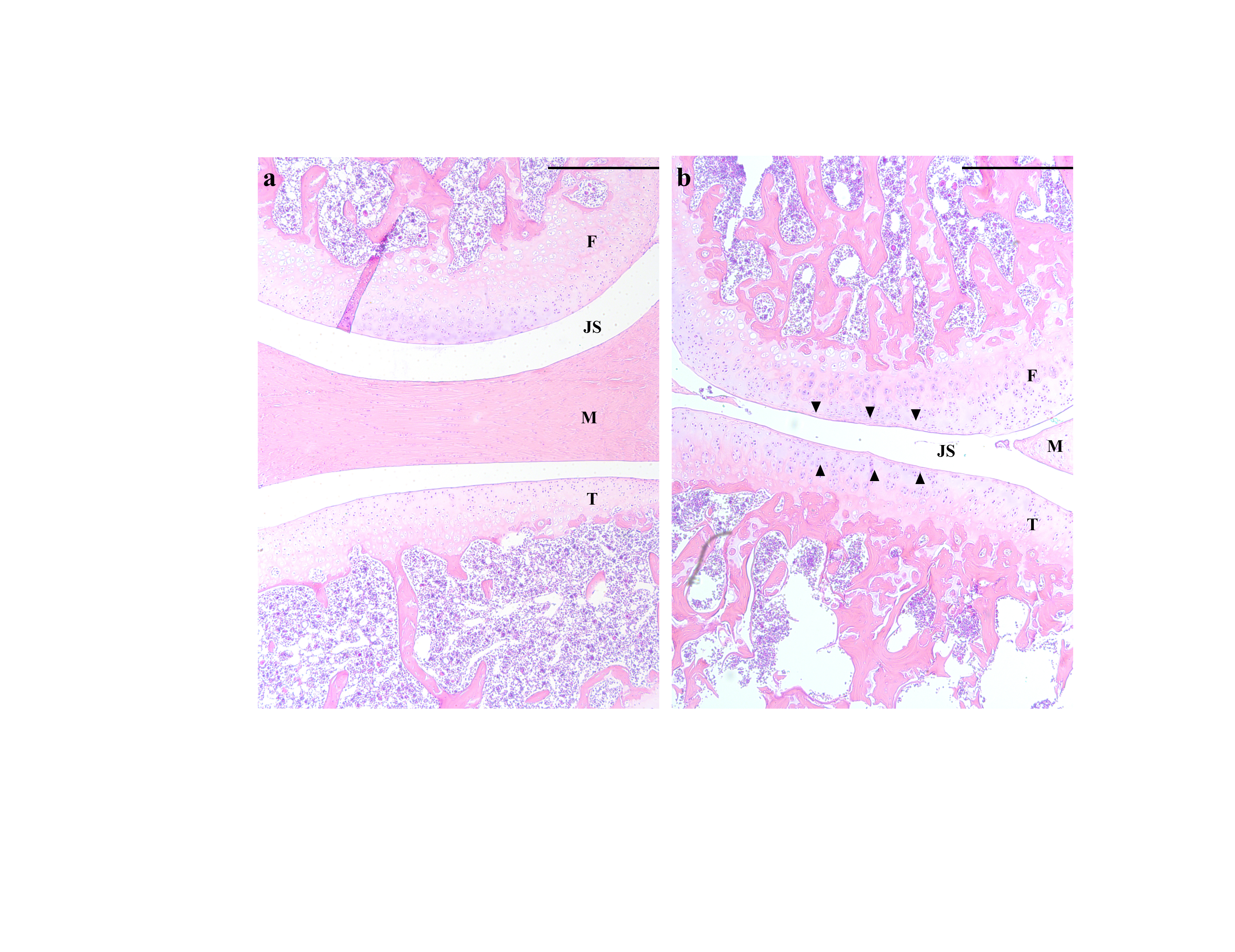

Supplement: Supplementary file 1 — Additional file 1: Figure S1. Histological features of the femoral and tibial cartilage surface at 2 weeks in Im-B and Im-NS groups. The cartilage surface was smooth in the Im-NS group (a), irregularity of the femoral and tibial cartilage surface was observed in Im-B group (b). F: Femur, S: Synovium, JS: Joint Space, M: Meniscus. The black arrowheads indicate cartilage irregularity. Scale bar = 500 μm. [file 12891_2020_3795_MOESM1_ESM.tif]

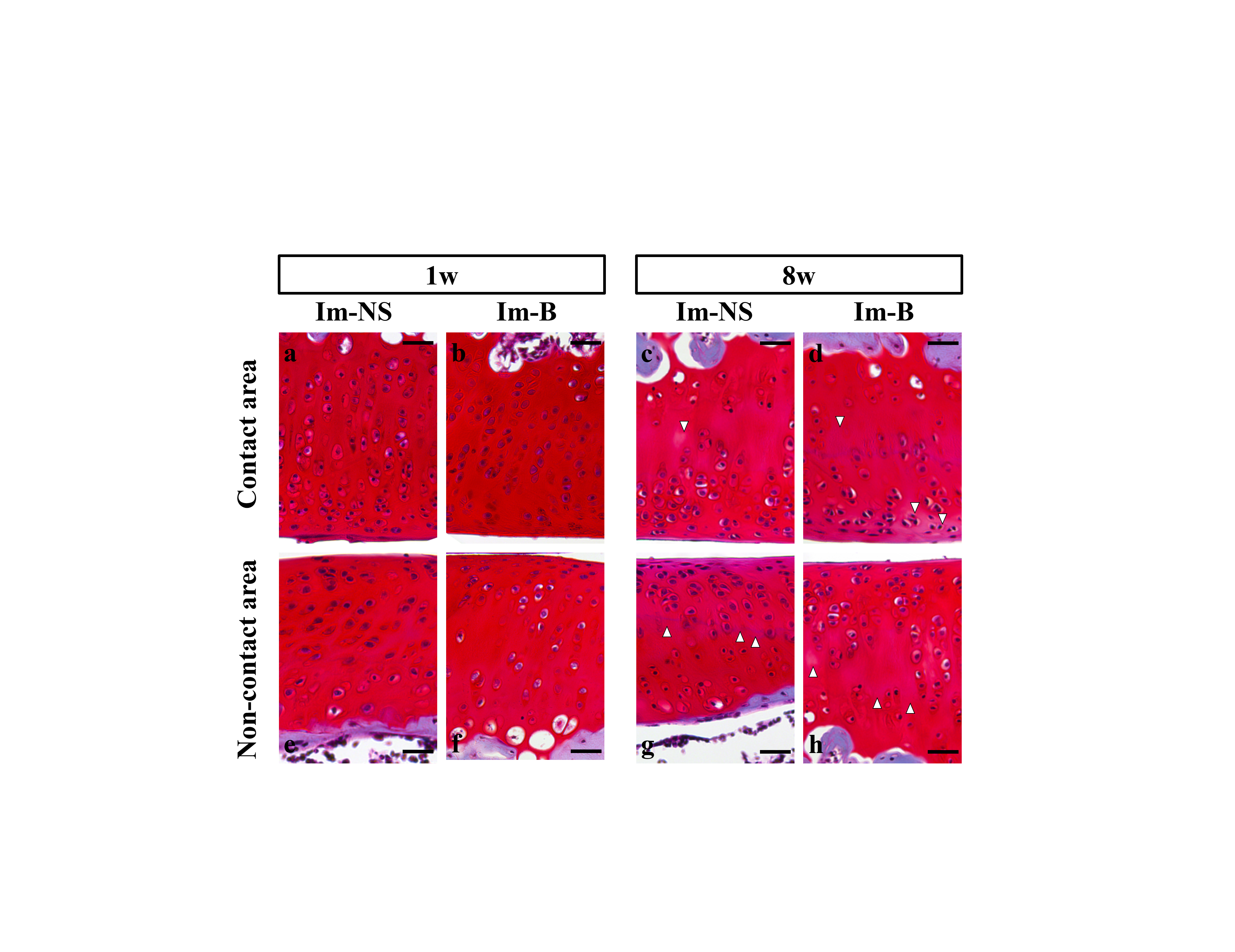

Supplement: Supplementary file 2 — Additional file 2: Figure S2. Safranin O staining of the femoral articular cartilage. The changes were observed after immobilization and intra-articular administration of blood (Im-B) or normal saline (Im-NS) at 1- and 8- weeks. The upper row (a-d) shows the changes in the cartilage in the contact area and the lower row (e-h) shows the changes in the cartilage in the non-contact area. A reduction in Safranin O staining intensity was not observed at 1 week in the contact and non-contact areas (a, b, e, and f). A reduction in staining intensity was observed at 8 weeks in the contact area, and the reduction in the Im-B group was more severe than that observed in the Im-NS group (c and d). A similar reduction in staining was observed in the non-contact area, and there were no significant differences between the Im-B and Im-NS groups (g and h). The white arrowheads indicate a reduction in staining intensity at the extracellular matrix. Scale bar = 50 μm. [file 12891_2020_3795_MOESM2_ESM.tif]

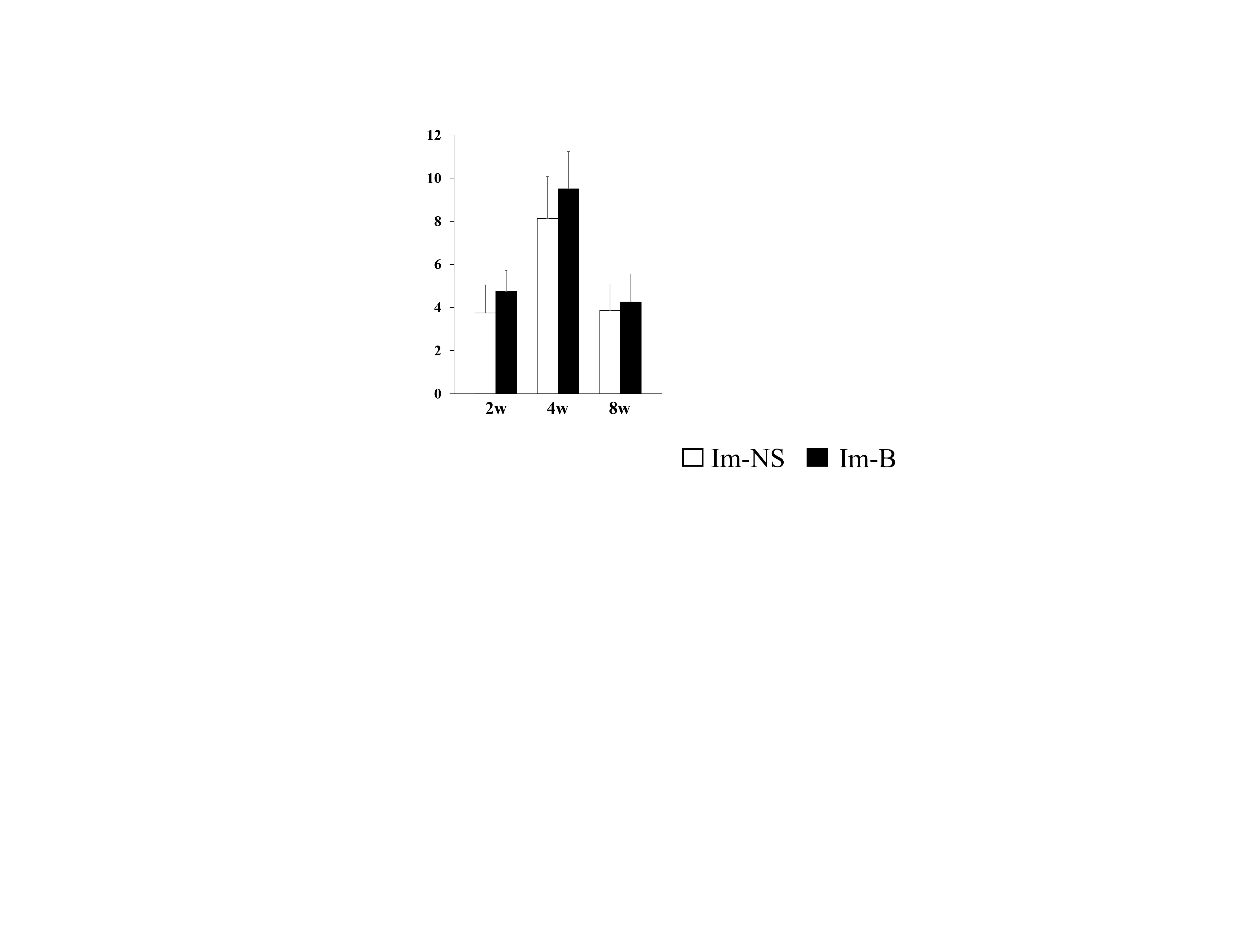

Supplement: Supplementary file 3 — Additional file 3: Figure S3. The histological scoring of synovitis from 2 to 8 weeks. The synovitis score of the Im-B group was slightly but not significantly higher than that of the Im-NS group at all periods. [file 12891_2020_3795_MOESM3_ESM.tif]

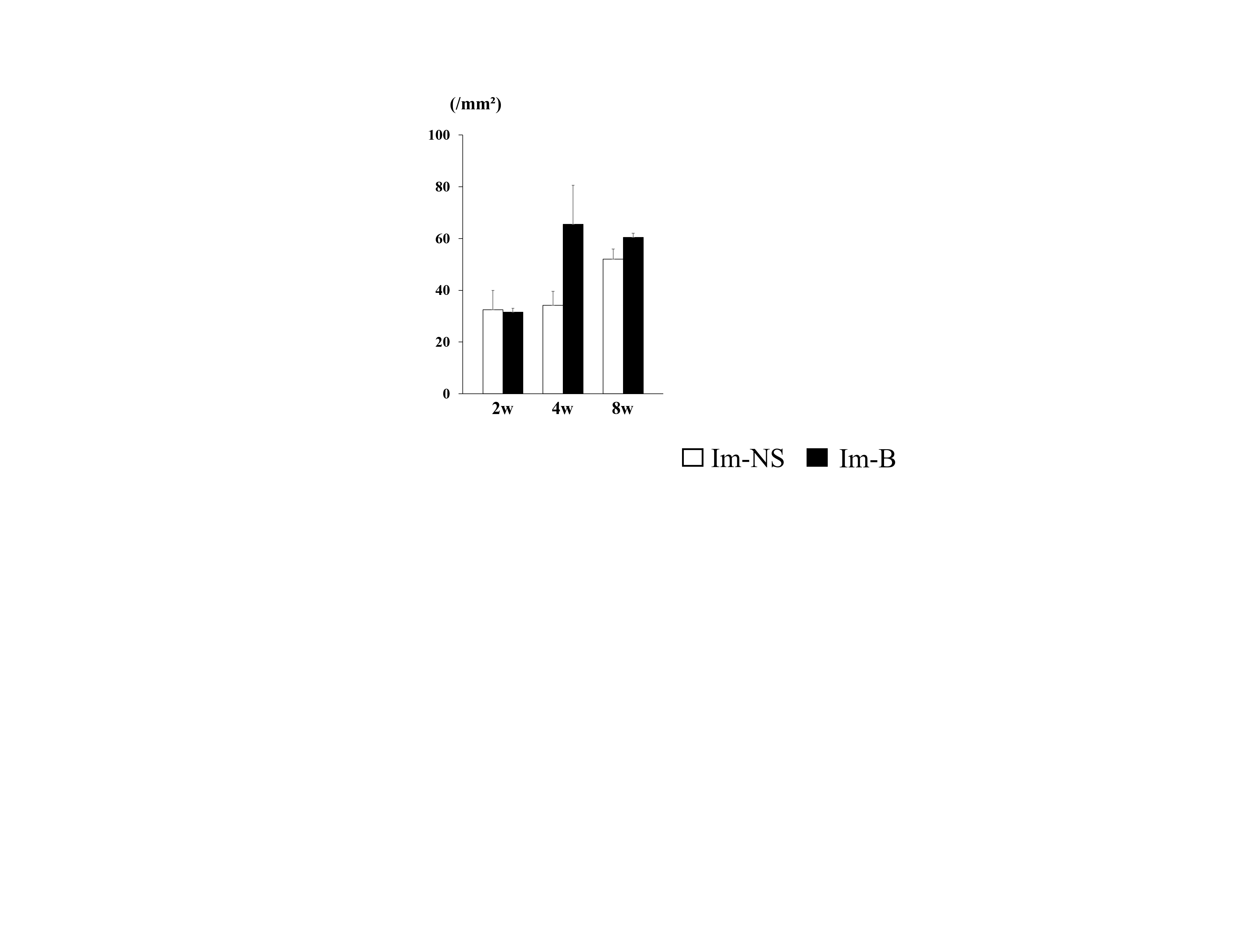

Supplement: Supplementary file 4 — Additional file 4: Figure S4. The number of CD68-positive cell from 2 to 8 weeks. The number of CD68-positive cells increased slightly but not significantly at 4 weeks in Im-B group and 8 weeks in Im-NS group. [file 12891_2020_3795_MOESM4_ESM.tif]
